# Expression of KIT Receptor Tyrosine Kinase in Endothelial Cells of Juvenile Brain Tumors

**DOI:** 10.1111/j.1750-3639.2009.00357.x

**Published:** 2010-07

**Authors:** Marjut Puputti, Olli Tynninen, Paula Pernilä, Marko Salmi, Sirpa Jalkanen, Anders Paetau, Harri Sihto, Heikki Joensuu

**Affiliations:** 1 Helsinki Biomedical Graduate SchoolHelsinki, Finland; 2 Laboratory of Molecular Oncology, Biomedicum HelsinkiHelsinki, Finland; 3 Department of Pathology, Helsinki University Central Hospital (HUSLAB) and University of HelsinkiHelsinki, Finland; 4 Department of Oncology, Helsinki University Central HospitalHelsinki, Finland; 5 Molecular Cancer Biology Program, University of Helsinki, Biomedicum HelsinkiFinland; 6 The Finnish Programme for Centres of Excellence in Research, Host Defence ResearchTurku, Finland; 7 MediCity Research Laboratory, University of TurkuTurku, Finland; 8 Department of Medical Biochemistry, University of TurkuTurku, Finland

**Keywords:** astrocytoma, endothelial cell, ependymoma, KIT, pediatric brain tumor, pilocytic astrocytoma, receptor tyrosine kinase

## Abstract

KIT receptor tyrosine kinase is expressed in tumor endothelial cells of adult glioblastomas, but its expression in pediatric brain tumor endothelial cells is unknown. We assessed expression of KIT, phosphorylated KIT, stem cell factor (SCF) and vascular endothelial growth factor receptor-2 (VEGFR-2) in 35 juvenile pilocytic astrocytomas and 49 other pediatric brain tumors using immunohistochemistry, and *KIT* messenger RNA (mRNA) using *in situ* hybridization. KIT and phospho-KIT were moderately or strongly expressed in tumor endothelia of 37% and 35% of pilocytic astrocytomas, respectively, whereas marked SCF and VEGFR-2 expression was uncommon. *KIT* mRNA was detected in tumor endothelial cells. Tumor endothelial cell KIT expression was strongly (*P* < 0.01) associated with endothelial cell phospho-KIT and SCF expression, and with tumor KIT (*P* = 0.0011) and VEGFR-2 expression (*P* = 0.022). KIT and phospho-KIT were present in endothelia of other pediatric brain tumors, notably ependymomas. Endothelial cell KIT expression was associated with a young age at diagnosis of pilocytic astrocytoma or ependymoma, and it was occasionally present in histologically normal tissue of the fetus and children. We conclude that KIT is commonly present in endothelial cells of juvenile brain tumors and thus may play a role in angiogenesis in these neoplasms.

## INTRODUCTION

KIT tyrosine kinase is a member of the platelet-derived growth factor receptor (PDGFR) subfamily III of receptor tyrosine kinases (17, 28). KIT binds specifically stem cell factor (SCF), which exists both as soluble and membrane-bound forms (1, 9, 15). Both KIT and SCF are expressed in a wide variety of tissues in the developing embryo and throughout the postnatal life, and are involved in numerous functions (3). In the nervous system KIT and SCF are expressed in the olfactory bulb, the cerebral cortex, the hippocampus, the cerebellum and in the spinal cord (8, 17, 27). Recombinant SCF induces neurite outgrowth from the dorsal root ganglia, where KIT-positive neurons can be detected already during the embryonic development and where its expression persists throughout the postnatal period (7). *KIT* mutations are associated with some adult cancers, notably gastrointestinal stromal tumors (GISTs; 6, 12, 18, 20).

Recent findings suggest that the SCF/KIT signaling plays an important role in angiogenesis of brain tumors and also of histologically normal tissues (24). SCF activates brain microvascular endothelial cells *in vitro* by enhancing proliferation, survival and migration of the endothelial cells. Tumor cells with the strongest SCF expression as well as normal SCF-expressing neurons are found predominantly within the infiltrating tumor border where angiogenesis is prominent (24). Moderate or strong expression of KIT and phosphorylated (activated) KIT have been detected in tumor endothelial cells of glioblastomas, but only infrequently in a few other histological types of human cancer (21). Signaling via KIT presumably enhances the umbilical vein endothelial cell tube formation, and increases endothelial cell survival *in vitro*(14). Taken together, these findings suggest that endothelial cell KIT may have a role in angiogenesis of brain tumors, and possibly of other types of human tumors and normal tissues as well.

Some pediatric tumors express KIT (2, 23), but little is known about expression or the role of endothelial cell KIT in pediatric brain tumors (21). In the present study we investigated expression of tumor and intratumoral vessel endothelial cell KIT, SCF and vascular endothelial cell growth factor receptor-2 (VEGFR-2) in pilocytic astrocytomas and other pediatric brain tumors.

## PATIENTS AND METHODS

### Tissue samples

Formalin-fixed, paraffin-embedded tissues were retrieved from 35 juvenile pilocytic astrocytomas diagnosed at the age of 18 or younger at the Helsinki University Central Hospital (HUCH), Helsinki, Finland, in 1985 to 2007, for analysis. The patients were not consecutive, but were selected at random. The median age at diagnosis was 10 years (range 0 to 18), and 18 (51%) were male. Most (62%) tumors were confined to the cerebellum and were treated with surgery. The median follow-up time of the patients was 5 years (range 0 to 18 years) after the diagnosis. Seven of the tumors recurred, but only one patient died during the follow-up.

Besides pilocytic astrocytomas, we examined 49 pediatric brain tumors of various histological types (11 ependymomas, 12 medulloblastomas, 7 dysembryoblastic neuroepithelial tumors, 4 gangliogliomas and 15 tumors of other histological type). These tumors were diagnosed within the same time period and at the same institute, and were also selected for analysis at random. The tumor diagnoses were reviewed by professional pathologists (O Tynninen, A Paetau) according to the World Health Organization (WHO) classification (13).

To examine vascular endothelial cell KIT expression during the fetal life and during childhood, histologically normal paraffin-embedded tissue samples from several organs were collected for analysis. The fetal samples were obtained from a fetus aborted at the gestational age of 18 weeks (n = 2) or from an autopsy performed due to a stillbirth at the gestational age of 23 weeks and 40 weeks (n = 4). Histologically normal tissue samples (n = 2) were also obtained from an autopsy of a young child who died at the age of 18 months, and from non-tumoral brain tissue of eight children, who underwent surgery to remove epileptogenic focal cortical dysplasia (age at the time of surgery was 3, 4, 8, 9, 10, 14 and 15 years).

The study protocol was approved by Institutional Review Boards of Helsinki and Turku University Hospitals, and a permission to use the tissue sections for the study was provided by the National Authority for Medicolegal Affairs of Finland.

### Immunohistochemistry

Five-micrometer whole tumor tissue sections were cut from representative formalin-fixed, paraffin-embedded tissue blocks. The tissue sections were stained for KIT, phosphorylated KIT, SCF, hypoxia-induced factor-1α (HIF-1α), and VEGFR-2 expression using immunohistochemistry as described elsewhere (21). KIT was stained with a rabbit polyclonal anti-CD117 antibody (dilution, 1:300; A 4502, DAKO, Glostrup, Denmark), phosphorylated KIT with a polyclonal rabbit anti-phospho-KIT antibody (#3391; 1:35; directed at the Tyr719 residue that mediates activation of the phosphatidyl inositiol 3-kinase (PI3K)/ Akt pathway; Cell Signaling Technology, Inc, Danvers, MA, USA), SCF with a monoclonal mouse anti-SCF antibody (1:200, hKL12; Acris Antibodies GmbH, Hiddenhausen, Germany), HIF-1α with a monoclonal mouse anti-HIF-1α antibody (1:100, H1alpha67, Ab-4; Neomarkers, Fremont, CA, USA), and VEGFR-2 with an epitope-specific rabbit anti-VEGFR-2 antibody (Flk-1 Ab-1, NeoMarkers, Lab Vision Corp., Fremont, CA, USA). The antibodies were diluted into a PowerVision preantibody blocking solution and were incubated with the sample. The binding of the primary antibody was detected with a Powervision+ Poly-HRP histostaining kit (DPVB+110DAB, ImmunoVision Technologies Co., Daly City, CA). The tissue sections were counterstained with hematoxylin & eosin.

**Figure 1:**
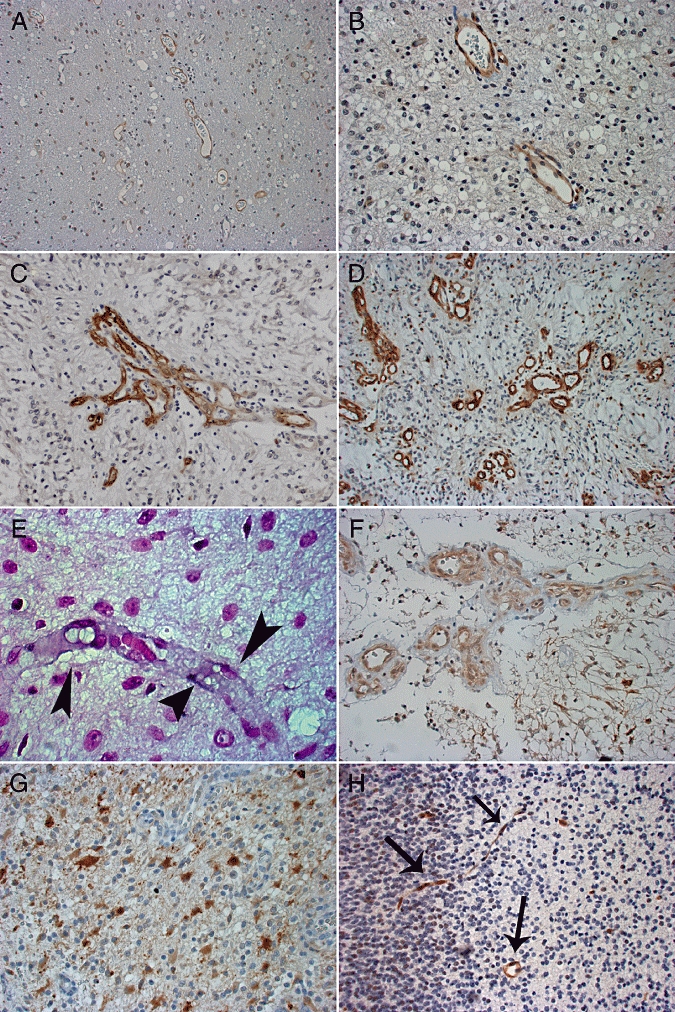
Faint (+, **A**), moderate (++, **B**) and strong (+++, **C**) expression of endothelial cell KIT in pediatric pilocytic astrocytomas (original magnification ×200, **A**; and ×400, **B,C**). A vascular proliferation with multiple lumens is shown in **C.** (**D**) Endothelial cell expression of phosphorylated KIT in pilocytic astrocytoma (+++; ×200). (**E**) *KIT* mRNA in endothelial cells (arrowheads) of pilocytic astrocytoma detected by *in situ* hybridization (×1000). (**F**) Faint (+) SCF expression in pilocytic astrocytoma (×400). (**G**) Moderate (++) expression of VEGFR-2 in astrocytoma tumor cells (×400). (**H**) Brain endothelial cells showing moderate expression of phosphorylated KIT in a 18-week-old fetus (×200, arrows).

Mast cells (which are strongly KIT-positive) were used as internal controls, and primary glioblastomas with a known expression of endothelial cell KIT, phosphorylated KIT, and tumor cell or perinecrotic expression of HIF-1α and SCF were used as positive controls in immunohistochemical staining (21). In addition, histologically normal bone marrow was used as a positive control in immunostaining for SCF and wound tissue for VEGFR-2. Immunostaining of the tumors was graded as negative (−), faintly positive (+), moderately positive (++) or strongly positive (+++) by a professional pathologist (O Tynninen) and one of the authors (M Puputti) using a consultation microscope (Nikon Eclipse E600, Nikon Instech Co., Ltd., Kanagawa, Japan). Tumor endothelial cell KIT expression was considered either negative (−), faintly positive (+) when 1% to 10% of the intratumoral vessels were positive, markedly positive (++) when 11% to 50% of the intratumoral vessels stained positively, and strongly positive when over 50% of the intratumoral vessels were positive (Figure 1A–C).

### mRNA *in situ* hybridization for *KIT*

Digoxigenin-labelled locked nucleic acid probes (LNA™ mRNA, Exiqon, Inc., Woburn, MA, USA) were designed against the *KIT* extracellular domain coding sequences 5′-gttgagaagagcctgtctggac-3′ and 5′-cttggcaggatctctaacaaacacat-3′. Efficacy of hybridization was controlled with a polyT(25)Vn probe (LNA™, Exiqon) against the poly(A) tail. *In situ* hybridization was carried out on whole deparaffinized tissue sections as described earlier with minor modifications (21). All reagents and instruments were either treated with diethyl pyrocarbonate (DEPC) or were made RNAse-free. The tissue sections were counterstained with 0.1% Nuclear Fast Red (Sigma-Aldrich Inc., St. Louis, MO, USA). The hybridization results were graded either as negative (−) or positive (+). Glioblastomas with a known endothelial cell KIT expression in the tumor vasculature served as positive controls (21).

### Chromogenic *in situ* hybridization (CISH)

CISH was performed as described elsewhere (25). In brief, 3-µm-thick whole tissue sections mounted on glass slides were deparaffinized and heat-pretreated in a temperature-controlled microwave oven. Bacterial artificial clone (BAC) probes for *KIT* were prepared as described elsewhere (11), and applied onto the slides. The sections were denatured in the presence of the BAC probes on a heat plate, and hybridization was expected to occur during an overnight incubation at +37°C. The signals were detected with a Spot-Light CISH detection kit (Zymed, Inc., South San Francisco, CA, USA) according to the manufacturer's protocol. Gene amplification was defined to be present when 6 or more signals, or clusters of signals, were detected in one nucleus in more than 10% of the counted nuclei. Endothelial cells within the tumor sections showed a diploid copy number in each case, and served as internal controls for hybridization.

### Statistical analysis

Frequency tables were analyzed using Fisher's exact test. When examining associations between tumor endothelial cell KIT, phosphorylated KIT and VEGFR-2, tumors with moderate (++) or strong (+++) expression in endothelial cells were grouped together and compared with those with either negative (−) or faint (+) expression. Tumor KIT, phospho-KIT and SCF expression was classified either as positive (+, ++ or +++) or negative (−) in statistical calculations. Non-normal distributions between the groups were compared using the Kruskal–Wallis or the Mann–Whitney test. Life tables were constructed using the Kaplan–Meier method, and survival between groups was compared using the log-rank test. The *P* values are two-sided.

## RESULTS

### Expression of KIT, SCF and VEGFR-2 in pilocytic astrocytomas

When endothelial cells (cells that lined the capillary lumens) stained positively for KIT, both delicate capillaries with flat endothelium and aggregations of vessels with hypertrophic and plump endothelial cells stained positively (Figure 1A–C). Moderate (++) or strong (+++) KIT expression was detected in tumor endothelial cells in 13 (37%) out of the 35 juvenile pilocytic astrocytomas, whereas in none of the cases the tumor cells expressed KIT at these levels (Figure 1A–C; Table 1). Vascular hyperplasia was present in 13 (37%) tumors (Figure 1C). Six (46%) of these tumors expressed KIT moderately strongly or strongly in endothelial cells of the vascular proliferations. Six (46%) out of the 13 tumors with vascular proliferations expressed KIT also in the flat endothelial cells compared with 7 (32%) out of the 22 tumors that lacked vascular hyperplasia (*P* = 0.48). Nonspecific staining of proteins in the vessel lumen was not detected, and abluminal cells (pericytes) did not stain for KIT.

**Table 1:** Expression of KIT, phosphorylated KIT, SCF, and VEGFR-2 in juvenile pilocytic astrocytomas. Abbreviations: N = number of cases; SCF = the stem cell factor; VEGFR-2 = vascular endothelial cell growth factor receptor-2.

Protein	Tumor endothelial cell expression N −/+/++/+++	Tumor cell Expression N −/+/++/+++
KIT	12/10/6/7	29/6/0/0
phospho-KIT	16/8/4/6*	30/4/0/0*
SCF	24/7/0/0**	24/6/1/0**
VEGFR-2	26/5/0/0**	10/15/6/0**

* Staining of one tumor was considered uninformative.

** Staining of four tumors were considered uninformative or adequate tissue was not available.

Staining for phosphorylated KIT was present both in delicate capillaries and hypertrophic vessels with no apparent association between phosphorylated KIT immunoexpression and microvessel morphology (Figure 1D). Phosphorylated KIT was moderately (++) or strongly (+++) expressed in endothelia of 10 (29%) tumors, but it was not expressed strongly in any of the cases in tumor cells. In contrast, moderate or strong SCF or VEGFR-2 expression was not detected in pilocytic astrocytoma endothelial cells [SCF was expressed weakly in 7 (23%) out of the 31 assessable tumors], whereas 6 (19%) tumors expressed VEGFR-2 moderately (++).

### *KIT* mRNA in endothelial cells

We used *in situ* hybridization to study presence of messenger RNA (mRNA) in the endothelial cells of 19 pilocytic astrocytomas. *KIT* mRNA was detected in the endothelial cells of 5 (26%) tumors. Four of these five tumors showed moderate or strong endothelial cell KIT expression in immunohistochemistry (Figure 1E).

### Copy number analysis of *KIT* gene

*KIT* gene copy number was assessed using CISH in 9 pilocytic astrocytomas. None of the tumors had amplification of the gene. Two tumors showed *KIT* aneuploidy (three to five *KIT* gene copies were present per one cell).

#### Associations between endothelial cell and tumor cell KIT, SCF and VEGFR-2 expression

Tumor endothelial cell KIT expression was highly significantly associated with endothelial cell phospho-KIT expression (*P* = 0.0009), suggesting that endothelial cell KIT was often activated. Endothelial cell KIT was also associated with endothelial cell SCF expression (*P* = 0.0017), but not with endothelial cell VEGFR-2 expression (*P* = 0.35, a representative staining shown in Figures 1F and G). Endothelial cell expression of phosphorylated KIT was associated with endothelial cell SCF expression (*P* = 0.0002), but neither endothelial cell phosphorylated KIT nor SCF expression was associated with endothelial cell VEGFR-2 expression (*P* = 0.62 and 0.57, respectively).

Pilocytic astrocytomas that expressed KIT in tumor cells expressed frequently KIT, phospho-KIT and SCF in the tumor microvessel endothelial cells as well, but not endothelial cell VEGFR-2 (Table 2). Tumor cell phospho-KIT expression was associated with endothelial cell KIT expression (*P* = 0.011), and tumor cell SCF expression with endothelial cell SCF expression (*P* = 0.029). Pilocytic astrocytomas that expressed VEGFR-2 expressed often endothelial cell KIT (*P* = 0.022) and phospho-KIT (*P* = 0.049), and tended to express SCF in the tumor endothelial cells (*P* = 0.077).

**Table 2:** Associations between tumor cell and tumor endothelial cell expression of KIT, phosphorylated KIT, SCF and VEGFR-2 in juvenile pilocytic astrocytomas. Abbreviations: SCF = the stem cell factor; VEGFR-2 = vascular endothelial cell growth factor receptor-2.

Tumor cell expression	Tumor endothelial cell expression			
	KIT	phospho-KIT	SCF	VEGFR-2
	P*	P*	P*	P*
KIT	0.0011	0.019	0.0078	1.0
phospho-KIT	0.011	0.07	0.41	1.0
SCF	0.65	0.33	0.029	0.21
VEGFR-2	0.022	0.049	0.077	0.24

* Fisher's exact test.

HIF-1α was evaluated in tumor cells and perinecrotic areas. HIF-1α was present only in 3 (9%) out of the 32 evaluated pilocytic astrocytomas, and its expression was faint (+) in all these cases. Two pilocytic astrocytomas contained necrotic areas, and HIF-1α was present in the perinecrotic tumor cells in both cases at a faint or moderate intensity.

#### Tumor endothelial cell KIT, age at diagnosis and survival

**Figure 2:**
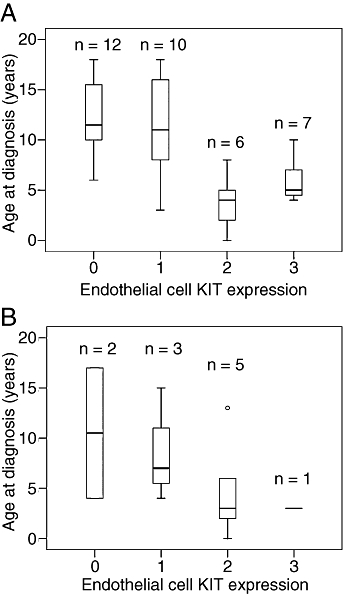
Association between age at diagnosis and endothelial cell KIT expression in pediatric pilocytic astrocytoma (**A**) and ependymoma (**B**). The box plots show the 10th, 25th, 50th (median), 75th and 90th percentiles.

A strong association was found between age at diagnosis and KIT expression in the endothelial cells of pilocytic astrocytomas. Patients diagnosed at a young age with pilocytic astrocytoma usually had moderate or strong tumor endothelial cell KIT expression, whereas those diagnosed at an older age had no or only faint KIT expression in the tumor endothelial cells (Kruskall–Wallis test *P* = 0.0012, Figure 2, upper panel; when analyzed moderate or strong expression vs. negative or faint expression using the Mann–Whitney test *P* = 0.0001). A similar association between age at diagnosis and endothelial cell phospho-KIT expression was also present (*P* = 0.048, Mann–Whitney test). Neither endothelial cell KIT nor phospho-KIT expression was significantly associated with recurrence-free survival in the present series (*P* = 0.84 and 0.09, respectively).

#### Tumor endothelial cell KIT, phospho-KIT and *KIT* mRNA in other histological types of pediatric brain tumors

**Figure 3:**
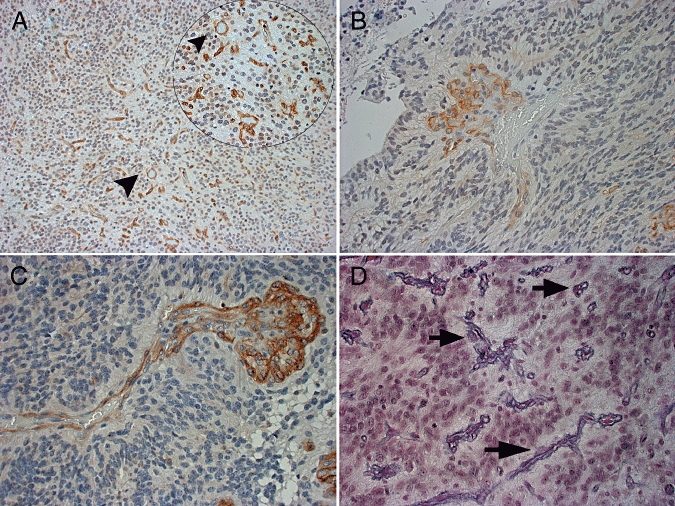
Ependymoma showing strong (+++) endothelial cell KIT expression in the tumor vessels (**A**; original magnification ×200, ×400 in inset) and in microvascular proliferations (**B**; ×200). (**C**) Ependymoma with strong expression of phosphorylated KIT in tumor endothelial cells (×400). *KIT* mRNA in tumor microvessel endothelial cells (**D**) detected by *in situ* hybridization (×400, arrows).

To investigate whether tumor endothelial cells might express KIT, SCF or VEGFR-2 also in other types of pediatric brain tumors than pilocytic astrocytoma, we examined 49 further tumors with immunohistochemistry (Tables 3 and 4). Marked (++ or +++) expression of KIT and phospho-KIT were frequently present in the endothelia of ependymomas [6 (55%) out of 11, and 5 (45%) out of 11, respectively]. Anaplastic ependymomas had glomeruloid microvascular proliferations resembling those seen in glioblastomas. Staining for KIT was positive in the endothelial cell cytoplasm of delicate capillaries (Figure 3A) and microvascular proliferations (Figure 3B). Occasional abluminal cells stained positively for KIT in the microvascular proliferations, although the staining intensity was lower than in endothelial cells lining the vessel lumens (Figure 3B). In anaplastic ependymomas endothelial cells and most of the abluminal cells of microvascular proliferations stained positively for phosphorylated KIT with uniform intensity (Figure 3C). Age at diagnosis and KIT expression in tumor endothelial cells had a similar association in ependymomas as in pilocytic astrocytomas, although this association was not statistically significant (*P* = 0.067, Mann–Whitney test; *P* = 0.33 Kruskal–Wallis test; Figure 2, lower panel).

**Table 3:** Tumor endothelial cell expression of KIT, phosphorylated KIT, SCF and VEGFR-2 in 84 pediatric brain tumors. Abbreviations: N = number of cases; SCF = stem cell factor; VEGFR-2 = vascular endothelial cell growth factor receptor-2; PNET = primitive neuroectodermal tumor; DNT = dysembryoblastic neuroepithelial tumor; SEGA = subependymal giant cell astrocytoma; PXA = pleomorphic xanthoastrocytoma.

Histological diagnosis	N	Age or median age (range)	KIT	phospho-KIT	SCF	VEGFR-2
			−/+/++/+++	−/+/++/+++	−/+/++/+++	−/+/++/+++
Pilocytic astrocytoma	35	10 (0–18)	12/10/6/7	16/8/4/6*	24/7/0/0*	26/5/0/0*
Ependymoma	11	4 (0–17)	2/3/5/1	1/5/2/3	9/2/0/0	9/1/1/0*
Medulloblastoma	12	9 (1–20)	7/4/1/0	4/5/2/0*	10/0/0/0*	3/4/0/0*
DNT	7	12 (6–17)	5/2/0/0	0/5/2/0	7/0/0/0	7/0/0/0*
Ganglioglioma	4	11.5 (8–19)	4/0/0/0	3/1/0/0	4/0/0/0	3/0/0/0*
Craniopharyngioma	3	17 (10–19)	1/0/0/0*	3/0/0/0	1/0/0/0*	0/0/0/0*
Astrocytoma	1	17 (17)	1/0/0/0	0/1/0/0	1/0/0/0	0/1/0/0
SEGA	2	12 (8–16)	2/0/0/0	1/1/0/0	2/0/0/0	2/0/0/0
Anaplastic astrocytoma	2	9 (7–11)	1/1/0/0	1/0/1/0	2/0/0/0	1/1/0/0
Chroid plexus papilloma	2	1.5 (0–3)	1/1/0/0	2/0/0/0	2/0/0/0	1/1/0/0
Oligodendroglioma	2	13 (7–19)	1/0/0/0*	1/1/0/0	2/0/0/0	2/0/0/0
Oligoastrocytoma	1	19	1/0/0/0	1/0/0/0	1/0/0/0	1/0/0/0
PXA	1	15	1/0/0/0	1/0/0/0	1/0/0/0	1/0/0/0
PNET	1	2	1/0/0/0	0/1/0/0	1/0/0/0	1/0/0/0
Total	84	10 (0–20)	40/21/12/8	34/28/11/9	67/9/0/0	56/13/1/0

* Immunostaining was not informative, or a representative sample tissue was not available in one or more cases.

**Table 4:** Tumor cell expression of KIT, phosphorylated KIT, SCF and VEGFR-2 in 84 pediatric brain tumors. Abbreviations: N = number of cases; SCF = stem cell factor; VEGFR-2 = vascular endothelial cell growth factor receptor-2; PNET = primitive neuroectodermal tumor; DNT = dysembryoblastic neuroepithelial tumor; SEGA = subependymal giant cell astrocytoma; PXA = pleomorphic xanthoastrocytoma.

Histological diagnosis	N	KIT	phospho-KIT	SCF	VEGFR-2
		−/+/++/+++	−/+/++/+++	−/+/++/+++	−/+/++/+++
Pilocytic astrocytoma	35	29/6/0/0	30/4/0/0*	24/6/1/0*	10/15/6/0*
Ependymoma	11	9/2/0/0	7/3/1/0	8/3/0/0	5/4/2/0
Medulloblastoma	12	7/5/0/0	8/3/0/0*	9/1/0/0*	6/1/0/0*
DNT	7	6/0/1/0	6/1/0/0	7/0/0/0	7/0/0/0
Ganglioglioma	4	2/2/0/0	4/0/0/0	2/2/0/0	3/0/0/0*
Craniopharyngioma	3	0/3/0/0	3/0/0/0	1/0/0/0*	3/0/0/0
Astrocytoma	1	1/0/0/0	1/0/0/0	1/0/0/0	1/0/0/0
SEGA	2	1/1/0/0	1/1/0/0	1/1/0/0	1/1/0/0
Anaplastic astrocytoma	2	2/0/0/0	2/0/0/0	2/0/0/0	0/2/0/0
Chroid plexus papilloma	2	0/2/0/0	2/0/0/0	2/0/0/0	2/0/0/0
Oligodendroglioma	2	0/1/0/0*	2/0/0/0	1/1/0/0	1/0/1/0
Oligoastrocytoma	1	1/0/0/0	1/0/0/0	1/0/0/0	1/0/0/0
PXA	1	1/0/0/0	1/0/0/0	1/0/0/0	0/0/0/0*
PNET	1	0/1/0/0	0/1/0/0	0/1/0/0	0/1/0/0
Total	84	59/23/1/0	68/13/1/0	60/15/1/0	40/24/9/0

* Immunostaining was not informative, or a representative tissue sample was not available in one or more cases.

When *KIT* mRNA expression was studied from seven ependymomas using mRNA *in situ* hybridization (4 of the 11 cases were uninformative), *KIT* mRNA was present in 6 (86%) cases in the tumor vessels. Four of the six ependymomas with *KIT* mRNA detected by *in situ* hybridization expressed endothelial cell KIT either moderately (++) or strongly (+++) in immunohistochemistry (Figure 3). Five ependymomas contained tumor necrosis, and HIF-1α was present in the perinecrotic tumor cells at a faint or moderate intensity in all these tumors. Four out of the five ependymomas with perinecrotic HIF-1α expression showed moderate (++) expression of KIT in tumor endothelial cells.

### KIT and phosphorylated KIT expression in normal pediatric and fetal tissues

Since tumor endothelial cell KIT and phosphorylated KIT tended to be more common in young children with pilocytic astrocytoma or ependymoma, we examined whether endothelial cell KIT expression might be present in the normal blood vessel endothelia during the fetal life and in young children. Strong (+++) endothelial cell KIT expression was detected in the lung alveolar wall capillaries of one 23-week-old fetus. Weak (+) KIT expression was present in endothelial cells of the small intestine in a 18-week-old fetus, and in endothelial cells of the brain, lymph nodes, adipose tissue and spleen of a 40-week-old fetus. Weak KIT expression was detected also in the brain endothelial cells of a 3-year-old child. Moderate (++) expression of phosphorylated KIT was present in the endothelial cells of the small intestine in the 18-week-old fetuses, and brain endothelial cells showed moderate expression of phosphorylated KIT in a 18-week-old fetus and strong expression in a 40-week-old fetus. When KIT and phosphorylated KIT expression were evaluated in the cerebral cortex tissues obtained form children at the age of 4, 8, 9, 10, 14 or 15 years, a small number of neurons (≤5%) were KIT-positive at each age, but no expression of phosphorylated KIT was detected, and the endothelial cells expressed KIT or phospho-KIT in none of the cases. Thus, KIT expression may occasionally occur in the endothelia of histologically normal tissues in the fetus and young children.

## DISCUSSION

We detected moderate or strongly KIT and phospho-KIT expression in endothelia of 37% and 35% of pilocytic astrocytomas using immunohistochemistry, and confirmed presence of *KIT* mRNA in tumor microvessel endothelial cells using *in situ* hybridization. Expression of KIT in pilocytic astrocytoma may be more frequent than this, as we did not carry out serial sectioning of the tumors in order to preserve tissue for possible future diagnostic needs. Presence of phosphorylated KIT in tumor endothelia suggests that endothelial KIT is frequently activated, and thus likely binds its ligand, although the level of SCF in the endothelial cells may sometimes be below the detection threshold of immunohistochemistry. KIT and phosphorylated KIT were present also in tumor endothelia of other types of pediatric brain tumors, such as ependymomas and medulloblastomas, suggesting that adult glioblastomas are not the only types of brain tumors that may express activated endothelial cell KIT receptor tyrosine kinase.

A growing body of evidence suggests that SCF signaling via the KIT receptor is angiogenic. Hypoxic stress activates the transcriptional activity of *SCF*, which enhances endothelial cell proliferation in the brain microvasculature both *in vivo* and *in vitro*(5, 24). Hypoxia upregulates HIF-1α and VEGF (19), and activation of KIT by SCF signaling may also result in expression of HIF-1α(10). Pilocytic astrocytomas do not usually show overt tissue necrosis, and the majority of the tumors did not express HIF-1α suggesting that tumor endothelial cell KIT expression may not have been HIF-1α and hypoxia-mediated. In line with this finding, marked expression of VEGFR-2, a major proangiogenic receptor, was rare in pilocytic astrocytomas and in most other types of pediatric brain tumors. Yet, tumor cell expression of VEGFR-2 was associated with endothelial cell expression of KIT, phosphorylated KIT and SCF. Taken together, these findings suggest that endothelial cell KIT may have a role in angiogenesis of pilocytic astrocytomas and some other types of juvenile brain tumors.

Intratumoral endothelial cells of pilocytic astrocytomas and ependymomas showed strong KIT expression when the tumors had been diagnosed at a young age (at an age less than 10 years), whereas endothelial cell KIT expression was usually faint or absent when the diagnosis had been made at an older age (Figure 2). This finding suggests that the molecular biology of tumor angiogenesis of young and older children might not be similar despite similar tumor histology. To investigate this unexpected finding further, we assessed KIT expression in endothelial cells of some histologically normal fetal tissues, and of brain and non-brain tissues of children. Histologically normal brain vessel endothelium expressed phosphorylated KIT both in an 18-week-old fetus and in a 40-week-old fetus, and KIT-positive vessels were present in the cerebral cortex of a 3-year-old child but not in the cerebral cortex tissues of older children aged 4 to 15 years. Although based on a small number of tissue samples available, these findings suggest that KIT and phosphorylated KIT are present in histologically normal fetal brain blood vessel endothelia, and that brain endothelial cell KIT expression may diminish with age. Thus, presence of KIT and activated KIT in brain tumor microvessels of young children might reflect KIT expression in the endothelial cells of normal brain tissue in the fetus and young children. KIT has been reported to be present in capillary endothelia of the fetal lungs, placenta and soft tissues (16). In accordance with these findings, fetal lung alveolar capillary endothelium showed strong KIT expression in the present study at the gestational age of 23 weeks, which was no longer present in a 40-week-old fetus.

Tumor endothelial cell KIT expression is frequent in adult glioblastomas (21). Glioblastomas are generally rapidly proliferating and highly lethal brain tumors, which typically contain tumor necrosis and have hypoxia-driven active tumor angiogenesis. Glioblastomas frequently show SCF and HIF-1α expression at the perinecrotic tumor regions (21). Endothelial cell KIT expression is frequent also in clear-cell renal cell carcinomas, where HIF-1α and VEGFR-mediated molecular mechanisms likely have an important role in tumor pathogenesis (22). Further studies are needed to investigate associations between tumor necrosis, histological grade and endothelial cell KIT expression in different types of human cancer, and the related molecular biological mechanisms.

Microvascular proliferations are considered a hallmark of glioblastomas. These peculiar vascular structures consist of endothelial cells lining the vessel lumen and abluminal cells expressing smooth muscle antigens but not endothelial markers (4, 26). In this series, anaplastic ependymomas showed microvascular proliferations that were morphologically similar to those found in glioblastomas. These proliferations expressed KIT and phosphorylated KIT both in endothelial and abluminal cells.

We conclude that KIT is commonly present in the endothelial cells of brain tumors in children. Endothelial cell KIT is often phosphorylated suggesting that it is activated. KIT and phosphorylated KIT can be detected also in fetal brain microvessel endothelial cells and in endothelial cells of histologically normal brain tissue of young children. In pilocytic astrocytomas, endothelial cell KIT expression is associated with a young age at the time of the diagnosis, and with tumor cell expression of KIT, phosphorylated KIT, and VEGFR-2. The SCF and KIT-mediated signaling may be important in tumor angiogenesis of pilocytic astrocytomas and some other types of juvenile brain tumors.
